# Abdominal Compartment Syndrome: Risk Factors, Diagnosis, and Current Therapy

**DOI:** 10.1155/2012/908169

**Published:** 2012-06-07

**Authors:** Gina M. Luckianow, Matthew Ellis, Deborah Governale, Lewis J. Kaplan

**Affiliations:** ^1^Yale-New Haven Hospital Surgical ICU, New Haven, CT 06520, USA; ^2^Section of Trauma, Surgical Critical Care and Surgical Emergencies, Department of Surgery, Yale University School of Medicine, 330 Cedar Street, BB-310, New Haven, CT 06520, USA; ^3^Fletcher Allen Health Care Emergency Department, Burlington, VT 05401, USA

## Abstract

Abdominal compartment syndrome's manifestations are difficult to definitively detect on physical examination alone. Therefore, objective criteria have been articulated that aid the bedside clinician in detecting intra-abdominal hypertension as well as the abdominal compartment syndrome to initiate prompt and potentially life-saving intervention. At-risk patient populations should be routinely monitored and tiered interventions should be undertaken as a team approach to management.

## 1. Introduction

The concepts of intra-abdominal hypertension (IAH) and abdominal compartment syndrome (ACS) are pervasive, but the objective criteria by which to diagnose each of these entities are often misunderstood [1]. IAH and ACS occur in both medical and surgical Intensive Care Units (ICU), the general ward, and may even occur the Emergency Department. Successful outcomes rely on early and accurate diagnosis combined with timely therapy [2–4]. Herein we describe these conditions, identify the at-risk patient populations, review diagnostic techniques as well as tiered medical management strategies, acute surgical therapy and long-term interventions to improve patient safety, optimize survival, and decrease morbidity. 

## 2. Epidemiology

Changes in fluid resuscitation paradigms, such as Early Goal Directed Therapy (EGDT) in the medical realm, and “damage control resuscitation” in the trauma realm, have increased patient survival [5, 6]. As a result of vigorous fluid resuscitation, however, each has also been associated with an unanticipated and undesired consequence—intra-abdominal hypertension and abdominal compartment syndrome (ACS). Given the detrimental effects of ACS (organ failure and death), heightened awareness surrounding the recognition of IAH and its progression to ACS, as well as the reporting of ACS, is paramount for optimal patient care. IAH is estimated to occur in 32.1% of ICU patients, and ACS has been reported in up to 4.2% of patients requiring critical care [7]. In order to identify each of these, one must be familiar with their definitions.

## 3. Definitions

According to the World Society of the Abdominal Compartment Syndrome (WSACS), ACS may be defined as sustained intra-abdominal pressure (IAP) of >20 mm Hg with the presence of an attributable organ failure [8]. While the WSACS has defined the parameters of ACS, it is important to delineate ACS from its predecessor, intra-abdominal hypertension. Absent from any disease processes, the average intra-abdominal pressure ranges from 5 to 7 mm Hg with a normal upper limit of 12 mm Hg [8]. Thus, a sustained IAP greater than 12 mm Hg, according to the WSACS, defines IAH. When evaluating the effects of IAH relative to organ perfusion, a more useful metric is abdominal perfusion pressure (APP). This is calculated by subtracting the IAP from mean arterial pressure (MAP). Hence, APP = MAP-IAP (normal = 60 mm Hg) [8].

## 4. At-Risk Patient Populations

Betro and Kaplan have described three patient populations that are most likely to develop ACS—the postoperative injured patient that experienced near-exsanguination, medical patients that have undergone large volume fluid resuscitation for severe sepsis, and the general surgical patient that required large volume resuscitation for an intra-abdominal catastrophe regardless of etiology. Patients with thermal injury also receive large volume fluid resuscitation and do so in the setting of capillary leak that may lead to rapid ascites accumulation and increased intra-abdominal pressure as well [9]. Major risk factors for the development of IAH and ensuing ACS are large volume resuscitation, massive transfusion protocol use, management with an open body cavity, core hypothermia, coagulopathy requiring component therapy, severe sepsis or septic shock, critical illness in the setting of cirrhosis or other liver failure accompanied by extant ascites, mechanical ventilation, and PEEP > 10 cm H_2_O pressure [8, 10]. In a similar fashion, extremity compartment syndromes can be aggravated by large volume fluid resuscitation as well, although these syndromes are not the topic of this paper.

## 5. Monitoring

Accurate and reliable monitoring of IAP is essential when IAH or ACS is suspected. The current “gold” standard for monitoring IAP is the intravesicular technique [10]. This method uses an indwelling urinary catheter, a pressure transducer, and a syringe or similar device, capable of infusing fluid. Advantages of this technique are its reliability, relative noninvasiveness, and elementary process. Using a closed (as opposed to an intermittently accessed or open) system presents no discernable risk of urinary tract infection [11]. In comparison, the gastric method is more cost-effective, correlates well with intravesical pressure, but may be acutely contraindicated in specific patient populations—gastric laceration repair, ileus or bowel obstruction with large volume gastric aspirate, and partial or total gastric resection [12]. Another means to measure IAP is via the inferior vena cava (IVC). A catheter is placed into the IVC through the right (easier) or left common femoral vein [13]. Although this monitoring technique offers continuous real-time data there are more risks associated with this method such as thrombosis, venous thromboembolism, venous or arterial laceration, femoral nerve injury, hematoma formation, pseudoaneurysm formation, and central line associated blood stream infection (CLABSI) [14].

 While the intravesicular technique is readily accepted as the “gold” standard, there is, however, controversy regarding the most appropriate amount of fluid to instill into the otherwise empty bladder. The typical amount of normal saline that is infused ranges from 25 cc to 50 cc [8, 10, 15]. The current recommendation by the WSACS is that no more than 25 cc be instilled into the bladder to avoid coaptation of the bladder walls around the measuring catheter [8, 10]. Greater volumes of bladder instillate degrade the fidelity of the measurement and may provide the clinician with a falsely elevated reading leading to inappropriate therapy [15]. Furthermore, correct placement of the transducer and correct timing of the measurement play a vital role in accuracy of the data. The transducer should be placed at the phlebostatic axis and the measurement taken at end expiration [8]. While muscular activity may affect the measurement, Betro and Kaplan suggest a temporary increase in sedation to reduce this confounder as opposed to the use of neuromuscular blockade [9]. A potential monitoring scheme is provided in Table 2.

## 6. Grades and Types of ACS

The WSACS has categorized IAH into grades, based upon worsening abdominal pressures (Table 3). Moreover, based on the etiology of the IAH, ACS can be defined as primary, secondary, or recurrent [8]. Primary ACS is predominantly associated with hemorrhage in either the peritoneal or retroperitoneal spaces and often accompanies injury. Secondary ACS occurs as a result of organ edema or ascites formation following a large volume fluid resuscitation and visceral reperfusion injury. This form of ACS is common in patients who have undergone large volume fluid therapy for sepsis resuscitation as well as burn management. It remains unknown whether in such circumstances secondary ACS is avoidable or an unavoidable accompaniment of the primary disease process. Recurrent ACS or “tertiary ACS,” as it was formerly known, occurs in lieu of prior medical or surgical intervention for primary or secondary ACS. This is typically seen in patients with recurrent hemorrhage or persistent accumulation of ascites [8, 10].

## 7. Pathophysiology

The pathophysiology of IAH has been described in many organ systems. The cardiac system is affected when IAPs are elevated because the external pressure exerted on the inferior vena cava leads to diminished venous return and thus decreased cardiac output [18]. The pulmonary system is affected largely because of pressure-induced cephalad displacement of the hemidiaphragms and creating a functional restriction of diaphragmatic excursion and pulmonary expansion. Patient's exhibit decreased respiratory compliance, hypoxemia (relative or absolute), decreased CO_2_ clearance, and distorted pulmonary flow characteristics [19]. Renal dysfunction manifesting as increased serum creatinine and oliguria is multifactorial. Extrinsic renal vein compression, as well as increased venous impedance from IVC compression cause decreased glomerular filtration, upregulation of antidiuretic hormone, and activation of the rennin-angiotensin system stimulating water conservation [10, 20]. The decreased cardiac output secondary to diminished venous return may also lead to acute tubular necrosis [18, 20]. One should note that rhabdomyolysis secondary to muscle crush injury may also lead to renal failure. In addition, the central neuraxis, liver, and gastrointestinal tract similarly suffer hypoperfusion, and when relieved, subsequent reperfusion injury manifested as visceral edema; the brain may be somewhat more protected by virtue of the properties of an intact blood-brain barrier.

## 8. Therapy

**Figure 1:**
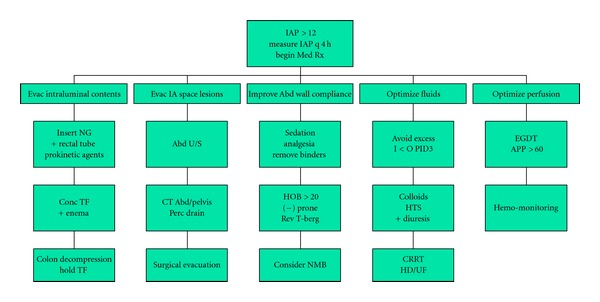
A tiered approach to IAH management (adapted from [16, 17]).

Both medical and surgical therapies have been described for IAH and ACS and need not be mutually exclusive. Medical management has been described in a tiered fashion and targets managing pressure-volume relationships in both the peritoneal space as well as the gastrointestinal tract therefore seeking to reduce intra-abdominal pressure ([16, 17], Figure 1). They more commonly provide only temporary IAP management although definitive therapy may be achieved for conditions such as ACS stemming from large volume gaseous colonic distension. In certain circumstances, neuromuscular blockade may be helpful in eliminating abdominal wall muscular tone to acutely manage or accurately measure IAP [16, 17]. However, this modality is surrounded by controversy and may not be appropriate for longer periods of time. No prospective randomized controlled studies exist to compare medical methods management to surgical techniques due to a lack of clinical equipoise regarding the need for definitive therapy when ACS is diagnosed. In that circumstance, surgical therapy provides definitive relief.

Nonetheless, since visceral edema and ascites are common accompaniments of secondary ACS, it is appropriate to review management options (see Table 1). Strategies to reduce intravascular volume include the use of potent loop diuretic agents such as furosemide, as well as and renal replacement therapy (RRT). Furosemide not only reduces intravascular volume but can also reduce bowel wall edema, leading to lower IAP. Acute kidney injury (AKI) as identified by the Acute Dialysis Quality Initiative and Acute Kidney Injury Network RIFLE criteria and oliguria are typically present in this population, despite volume resuscitation and total body salt and water excess [21, 22]. As such, given the hemodynamic lability of those with IAP and incipient ACS, RRT may have a role in managing metabolic clearance as well as total body solute load. The reader should note that RRT is generally not required for the management of all but late-stage AKI. Relatedly, appropriate vasopressor use as an adjunct in managing mean arterial pressure may serve to limit the total volume of fluids that are utilized in resuscitation. Management using a fluid “cap” has been described in one series and was associated with a reduced incidence of IAH and ACS [23]. Additionally, other strategies such as pulse pressure variation, pulse power, esophageal Doppler analysis, or transthoracic bioimpedance techniques may serve as guides to judicious fluid administration [24, 25]. 

Reductions in small and large bowel volume may also decrease IAP. The choice of technique depends on the cause of the luminal distension. If there is fecal impaction rectal lavage may be helpful especially when combined with an oral cathartic such as a polyethylene-glycol lavage solution. Gaseous gastric or liquid distension may be managed with oro- or nasogastric tube insertion and aspiration. This has shown to be helpful in small bowel obstruction management in some (but not all) patient series [26]. Nasogastric drainage is logically most effective in managing intra-abdominal pressure when there is gastric distension. Finally, gastro- and colonic-prokinetic agents, such as metoclopramide and erythromycin ethylsuccinate (EES) may increase intestinal transit time and evacuate luminal contents; some controversy surrounds the efficacy of these agents so they are not universally recommended. In those without cardiac disease, neostigmine may be particularly helpful when there is gaseous colonic distension as in Ogilvie's Syndrome [27].

Increasingly invasive, percutaneous catheter drainage (with indwelling catheter placement) or large volume paracentesis is another strategy to achieve intra-abdominal volume reduction when the increased IAP or ACS is due to massive ascites related to secondary ACS [28–30]. Technical difficulties may arise with such methods including catheter kinking, malposition, secondary ascites infection, and perforation of bowel or other intra-abdominal structures including major vascular domains.

As noted earlier, other organ systems are affected by the distorted physiology that accompanies IAH leading to ACS. As IAP increases and pulmonary compliance decreases, oxygenation and ventilation are progressively compromised. No single ventilator management strategy will overcome the untoward effects of rising IAP and ACS. However, as a temporizing measure, either changing to a pressure cycled mode with a prolonged inspiratory time, or using Airway Pressure Release Ventilation (APRV), a modified form of CPAP that uses high pressures to aid in alveolar recruitment, are two methods to temporize oxygenation and ventilation failure until definitive relief from ACS may be accomplished. APRV in particular has been shown to increase pulmonary flow and increase cardiac output as a result of decreasing pulmonary hypoxic vasoconstriction [31].

As noted above, when medical management fails, or ACS is present, surgical management is appropriate and generally consists of decompressive laparotomy; minimally invasive methods have been described but are not the standard or care at present for primary or recurrent ACS. Decompressive laparotomy may be performed in the OR or the ICU and affords rapid relief from ACS [32]. Such a laparotomy is generally followed by a temporary abdominal wall closure (of which there are many successful varieties) to create a functionally enlarged peritoneal space to decrease the likelihood of recurrent IAH and ACS. The reader should note that neither a homemade “VAC” (vacuum assisted closure), nor the proprietary KCI device prevents ACS from recurring. Thus, the bedside clinician should be vigilant and routinely monitor such patients for rising IAP.

## 9. Postdecompression Care

While decompression may be done at virtually any facility where there is a surgeon, the aftercare may be labor and resource intensive for small facilities. Therefore, postdecompression consideration should be made prior to transferring such patients to a facility capable of rendering such care. Generally an appropriate facility will be a level II or level I trauma facility where there is an embedded and dedicated ICU team as well as an in-house anesthesiologist and often an in-house surgeon. These patients require at least one and frequently several reexplorations prior to definitive closure, many of which may be accomplished in the ICU under deep sedation. Repeated temporary abdominal wall closure is generally required prior to closure [33]. For those who are unable to be closed at their first reexploration, many management options exist and may be categorized into (1) techniques to stretch fascia, (2) fascial spanning techniques, (3) component separation techniques with or without an underlay of mesh (biologic or synthetic), and (4) temporary mesh followed by a split thickness skin graft.

Devices that are sutured to fascia and use a hook-and-loop closure method progressively draw the edges of separated fascia together to facilitate primary closure. The Wittmann patch is one such device and has been used to good effect after injury [34]. Fascial spanning techniques may rely on synthetic mesh such as polypropylene or expanded polytetrafluoroethylene (Gortex), but have fallen out of favor due to unfavorable infection and recurrence rates. Spanning with acellular human dermis (AlloDerm) has similarly fallen out of favor due to gapping of the product over time creating a lax abdominal wall with deleterious cosmetic and functional results [35]. Instead, primary fascial closure coupled with an underlay of acellular human or porcine dermis (Strattice) has excellent results. Clinical results buttress the underlay technique as an ideal manner in which to use biologic mesh [36]. A multitude of other meshes are similarly available and may be used in an identical underlay fashion with good results as well. 

A component separation of parts technique may enable fascia to fascia approximation as well as support an underlay of biologic or synthetic mesh. However, the operative time is increased as is blood loss. Furthermore, this technique may not be appropriate for heavily contaminated spaces as it will open unexposed tissue planes to a large bacterial innoculum. Of course, implanting any permanent mesh is ill advised in the setting of heavy contamination, active infection, and perhaps even inadvertent enterotomy [37]. In general, component separation techniques are typically reserved for later reconstructive efforts in those who were unable to be closed during their initial hospitalization.

For those who remain open during their index hospitalization due to visceral edema or fistula formation, placing an absorbable mesh such as polyglycolic acid (Vicryl mesh) that hydrolyzes over time while the underling viscera establish a bed of granulation tissue is a commonly employed salvage strategy. Once there is a covering bed of granulation tissue, a STSG may be placed over the granulation bed and secured in place with a VAC device. There is no need to remove any residual absorbable mesh provided that the entirety of the mesh is embedded in granulation tissue; nonadherent or nonincorporated mesh should be excised as part of the tissue bed preparation at the time of skin grafting. Of course, for those who have already adhesed their visceral block, no absorbable mesh need to be placed and the viscera may be directly skin grafted once there is an appropriate granulation bed. The lack of an absorbable mesh often renders skin graft removal for later reconstruction more difficult in one author's view (LJK), but the technical difficulty is not insurmountable.

## 10. Abdominal Wall Reconstruction

The timing of abdominal wall reconstruction is generally 6 to 12 months after the last operation to allow inflammation to subside. It is unclear whether inflammation regression may be accurately tracked by following c-reactive protein levels, and study is underway to answer this question. Nonetheless, a commonly used surrogate is the “pinch test” that assess whether a skin graft may be rolled between the examiner's fingers instead of remaining densely adherent to the underlying viscera. Generally, if the pinch test is negative by 12 months after the last abdominal operation, more time will not be helpful. Reconstruction involves removing the skin graft, lysing adhesions, restoring gastrointestinal continuity if there is an ostomy, and then achieving fascia-to-fascia closure. Component separation of parts, biologic mesh underlay, pedicled rotational flaps (tensor fascia lata), and even free tissue transfers (latissimus dorsi muscle most commonly) using microvascular techniques have all been described to restore abdominal wall integrity [35]. Success is enhanced by reductions in visceral adipose mass, tobacco cessation, and repletion of lean body mass losses to support wound healing. Careful attention to providing multivitamins, B_12_, vitamin C, and supplemental zinc are all aids in wound healing following abdominal wall reconstruction; patients on therapeutic glucocorticoids should also receive vitamin A to mitigate against steroid-induced wound failure [38]. While the occurrence of an inadvertent enterotomy during ventral hernia repair is strongly associated with postoperative surgical site infection and hernia recurrence, whether colostomy or ileostomy takedown should occur as a staged procedure to precede restoration of abdominal wall integrity remains unknown [37]. 

## 11. Long-Term Results

The major complications of the surgical management of ACS have been well chronicled and principally grouped into mechanical, infectious, resource utilization, and quality of life. The “natural history” of injured patients suffering form ACS and receiving surgical therapy has been identified by Fischer et al. Readmission, reoperation, enteroatmospheric fistula, long-term mechanical ventilation, and tracheostomy, a wide variety of infections (pulmonary, surgical site, urinary tract, and CLABSI) as well as rehabilitation or skilled nursing facility placement are key events that occur with substantial frequency [39]. Nonetheless, outcomes after intervention were good with a large proportion returning to an independent functional status. A major factor influencing outcome is quality of life (QOL). For this patient population, the decrement in QOL stemming from having an ostomy or a planned ventral hernia has been characterized but has broad overlap with QOL decreases associated with prolonged ICU care [40]. Decreases in QOL have been noted for those having prolonged ICU care regardless of cause. It is likely that this is an underreported complication due to a lack of investigation as well as imprecise tools and the well-described transient nature of injured patients. US return to work rates are similarly difficult to interpret due to the relatively high unemployment rates of inner city trauma patients [41].

## 12. Future Directions

At present, we are behind the curve in diagnosing ACS. Ideally, we would want to be able to diagnose incipient ACS to intervene prior to the onset of end-organ damage. All too often, AKI persist and may progress to acute renal failure after what is thought to be expedient abdominal decompression. As in many other organ systems, having a readily identifiable biomarker of incipient organ system damage would be ideal. Such a marker would be elevated prior to the onset of renal tubular injury or increase in serum creatinine. It would ideally be able to differentiate the above from the decreased glomerular filtration rate and renal blood flow that accompanies hypovolemia. No such marker yet exists, recognizing that AKI does not stem from hypoperfusion and is instead a toxic phenomenon [42]. It may be that a panel of multiple biomarkers addressing multiple organ systems will be able to do so. Relatedly, such a panel has been articulated for the onset of funisitis, so there is belief that such a panel may be identified for ACS-associated organ injury as well [43].

In a complementary fashion, improved understanding of the human genome may be able to identify high-risk patients based on their genomics or proteomics. We remain in the infancy of understanding how genomics and proteomics influence the response to injury and critical illness. Nonetheless, such investigations have occurred in patients undergoing thoracotomy as a means of understanding persistent postthoracotomy pain [44]. Patients with a particular genetic profile are at higher risk for postthoracotomy pain persistence and as such fare better with specific anesthetic technique modifications. This peri-operative period of interaction between a patient's specific genetic profile and therapeutic intervention has been termed the “perioptome” and may serve as a template for future diagnostic and therapeutic interventions in a similar vein [44].

Unexplored is the occurrence of organ specific compartment syndromes. By way of example, patients with blunt renal, splenic, or hepatic injury with an intact capsule may have excessive organ pressure from a large and undrained hematoma. In particular, intrarenal hemorrhage may create the greatest risk as the kidney is bounded by a strong and well-developed fascial envelope—Gerota's fascia. We do not currently measure intraorgan pressures, and in general do not explore such injures, a byproduct of which is fascial compartment decompression. At present, we have no means of readily assessing for organ-specific compartment syndromes. Another such organ is the lungs. We are continually apprised of a plethora of airway pressure measurements, but when they are elevated, our primary interventions include gas delivery and volume manipulations as well as drainage of pleural gas or fluid collections. Only when there is ACS is surgical intervention undertaken. While thoracic compartment syndrome has been described, it is ONLY after the chest is already open and then unable to be closed [45]. To date, there are no reports of thoracic cage decompression for compartment syndrome and may represent a missed opportunity for intervention.

## 13. Conclusions

Intra-abdominal hypertension and the abdominal compartment syndrome are important to recognize and diagnose. Routine bladder pressure monitoring is the key to appropriately enacting medical and surgical therapy designed to mitigate against the untoward effects of increased intra-abdominal pressure. While labor intensive and resource consuming, surgical therapy for ACS is successful and restores the majority of patients to an independent functional status. Multiple adjunctive therapies are often required to optimize outcome in this challenging subset of critically ill patients. As such, early transfer to an appropriate center should be considered with rising intra-abdominal pressures or after the initial decompressive laparotomy. A multidisciplinary approach is generally required to meet the intensive care, general ward, convalescent, and reconstructive needs of these patients. Further work is needed to define and deploy strategies to mitigate against decrements in quality of life following the critical care of the patient with an open abdomen.

## Figures and Tables

**Table 1:** Common surgical options for ACS management.

Initial management of ACS	
Minimally invasive	
Percutaneous aspiration	
Colonoscopic decompression of colonic distension	
Invasive	
Initial or relaparotomy	

Open abdomen management (short-term; able to close primarily)	

Vacuum assisted closure (proprietary of home-made)	
Hook and Loop closure device	

Open abdomen management (long-term; unable to close primarily)	

Polyglycolic acid mesh and split-thickness skin graft (STSG)	
STSG without underlying absorbable mesh	
Component separation of parts with biologic mesh underlay (rare)	
Fascial relaxing incisions with spanning mesh (prosthetic or biologic)	
*Caution: biologic may relax and gap when placed in spanning position	

Reconstruction	

Component separation of parts with biologic mesh underlay (common)	
Primary closure + fascial relaxing incisions + biologic mesh underlay (less common)	
Free muscle flap + biologic mesh underlay (rare)	

**Table 2:** Bladder pressure monitoring guideline.

Patients covered	
All ICU patients at risk for intra-abdominal hypertension.	

*Risk identifiers for increased intra-abdominal pressure (IAP)*:	

(1) Damage control laparotomy.	
(2) Intra-abdominal procedure *in conjunction with* large volume resuscitation (>10 liters crystalloid equivalent), or Coagulopathy requiring correction with the massive transfusion protocol, or Large volume blood component therapy (PRBC > 10 units, or FFP > 8 units).	
(3) Severe sepsis or septic shock.	
(4) Open body cavity.	
(5) Core hypothermia.	
(6) Cirrhosis or liver failure with ascites.	
(7) Mechanical ventilation with PEEP > 10 cm H_2_O pressure (intrinsic or extrinsic).	
(8) Physician discretion.	

*Definitions*	

(1) Intra-abdominal hypertension: IAP > 12 mm Hg.	
(2) Abdominal compartment syndrome: a clinical syndrome resulting from increased IAP > 20 mm Hg coupled with an attributable organ failure manifested as increased peak airway pressure, oliguria, metabolic acidosis, decreased cardiac performance (mean arterial pressure, cardiac output, SvO_2_), decreased abdominal perfusion pressure, and decreased mentation. The ACS is commonly associated with IAP > 20 mm Hg but may occur at lower pressures as well based on individual patient characteristics.	
(3) Abdominal perfusion pressure (APP): Mean arterial pressure (MAP)-(IAP); Normal APP > 60 mm Hg	

*Guideline*	

(1) On admission to the ICU, patients will be evaluated by the bedside nurse and the physician team for risk identifiers for increased IAP.	
(2) Patients who are identified at-risk will be monitored by bladder pressure measurements according to the following schedule:	
(a) On arrival to the SICU.	
(b) Every 2 hours for the first 8 hours.	
(c) Every 4 hours for the next 8 hours.	
**(**d) Every 8 hours for the next 24 hours.	
(3) The ICU bedside team (physician and nursing) will decide on the frequency on IAP measurements after the first 24 hours of monitoring.	
(4) The physician team will be notified of all bladder pressure measurements >12 mm Hg and abdominal perfusion pressures < 60 mm Hg.	
(5) These values will be recorded on the nursing record.	

**Table 3:** Grades of intra-abdominal hypertension.

Grade	Intra-abdominal pressure
I	12–15 mm Hg
II	16–20 mm Hg
III	21–25 mm Hg
IV	>25 mm Hg
